# Spatio‐temporal assessment of nearshore fish communities in a temperate estuary using functional and community metrics for restoration and management

**DOI:** 10.1111/jfb.70171

**Published:** 2025-08-07

**Authors:** Zoe Morrall, George P. Balchin, Ian Hendy, Jen Lewis, Dominic Longley, Nick Rogers, Gordon Watson, Joanne Preston

**Affiliations:** ^1^ Institute of Marine Sciences, School of Environment and Life Sciences University of Portsmouth Portsmouth UK; ^2^ Sussex Inshore Fisheries and Conservation Authority Shoreham‐by‐Sea UK; ^3^ Environment Agency Romsey UK

**Keywords:** estuarine fish communities, fish estuarine association Score (FEAS), functional guilds, long‐term monitoring, nursery habitats

## Abstract

Understanding the structure and function of estuarine fish communities is essential for guiding ecosystem‐based management and restoration. This study investigated long‐term patterns in fish community composition, abundance, species richness and estuarine use across 15 nearshore sites within three estuaries and the Isle of Wight, located in the Solent, a large temperate estuarine system in southern England. Using a 12‐year dataset (2007–2018) of biannual seine net surveys, we applied traditional community metrics alongside functional guild classifications and Fish Estuarine Association Scores (FEAS) to assess spatio‐temporal variation and estuarine dependency. A total of 55 species were recorded, with six species (*Atherina presbyter, Dicentrarchus labrax*, *Pomatoschistus microps* and *Chelon auratus* and the family Clupeidae) accounting for 96% of individuals. Marine migrants and estuarine residents dominated the assemblage, indicating strong connectivity between estuarine and coastal habitats. While fish abundance declined significantly over time, species richness and community composition varied across seasons, tidal states and spatial scales. Sites and catchments differed markedly in FEAS, with some areas supporting species more dependent on estuarine habitats. These results highlight the importance of multisite, seasonal monitoring and the value of trait‐based metrics in identifying nursery habitats and guiding restoration. The FEAS approach, applied retrospectively to historical data, offers a practical framework for setting ecological baselines and prioritising functionally important estuarine areas under real‐world monitoring constraints.

## 1 INTRODUCTION

Coastal and estuarine systems are among the most productive habitats in the marine environment (Kennish, 2023; Stamp et al., 2022). These areas are essential fish habitats, providing critical nursery functions, supporting the early life stages of many fish species and contributing to adult fish stocks and biomass (Nagelkerken et al., 2015; Nodo et al., 2023). Within these systems, oyster reefs, seagrass meadows, mudflats and saltmarsh form a dynamic mosaic that provide shelter and feeding opportunities for juvenile and nearshore fish (Beumer et al., 2002; Sheaves, 2009a). These mosaics play a critical role by enhancing the trophic energy flow both within and across habitat boundaries (Sheaves, 2009b). Estuarine environments in particular often support higher densities of juvenile fish compared to adjacent nearshore marine areas (Nodo et al., 2023), highlighting the importance of understanding habitat–fish relationships to inform ecosystem‐based management strategies (Meynecke et al., 2008). As such, the connectivity and diversity of estuarine habitats are key to sustaining fish populations and their broader ecological function (Nagelkerken et al., 2015; Nodo et al., 2023).

Despite their ecological importance, estuarine habitats are often under‐represented in fisheries research, which has traditionally focused on offshore stocks and commercially targeted species (Moore et al., 2024; Thurstan et al., 2010). This has created knowledge gaps in nearshore systems, particularly concerning juvenile and estuarine‐resident fish assemblages and the environmental or spatial drivers of their variability. Estuarine systems are naturally dynamic, influenced by tidal cycles, seasonal patterns and longer‐term climate influences (Kinard et al., 2021; Lourenço et al., 2023; Whitfield, 2021). These natural processes interact with a range anthropogenic pressures, including habitat modification, pollution and urban development (Ahmed & Tamim, 2025; Crain et al., 2009), making it difficult to distinguish natural variability from anthropogenic impacts. Spatial variation adds further complexity because different estuarine habits support distinct assemblages and ecological function (Sheaves, 2009b; Woodland et al., 2019).

As the ecological condition of many estuarine habitats continues to decline under these combined pressures, restoration has become an urgent global priority (Cloern et al., 2016; Oliveira et al., 2024; Waltham et al., 2021). However, restoration efforts are often constrained by the difficulty of defining reference conditions. Restoration commonly aims to recover historical ecological sates (SER, 2004), but shifting baselines and limited long‐term data make this challenging (Estes et al., 2011; McClenachan et al., 2024; Pauly, 1995; Shackelford et al., 2024). Over time, degraded states may be accepted as normal (Alleway et al., 2023), resulting in lowered restoration targets (Hallett et al., 2013). Long‐term datasets are therefore key in tracking ecological change and subsequently restoration progress (Magurran et al., 2010). Whilst environmental factors such as tidal flow, salinity, temperature and oxygen all shape fish communities (Arevalo et al., 2023; González‐Sansón et al., 2022; Hagan & Able, 2003; Huntsman et al., 2023; Lai et al., 2024), such data are often lacking in historical records, which can reduce the ability to attribute changes to environmental drivers.

To address this, functional ecological metrics offer a practical means of assessing fish community structure and estuarine use across spatial and temporal scales in the absence of environmental data. Two complementary approaches are the Estuarine Use Functional Guild (EUFG) classification and the Fish Estuary Association Score (FEAS). EUFG groups species based on life‐history strategies and patterns of estuarine use (Elliott et al., 2007), whilst FEAS assigns each species a score from 1 to 5, reflecting its degree of estuarine dependency (Froese & Pauly, 2019; Harrison & Whitfield, 2021). As these are trait‐based metrics, they can be retrospectively applied to historical fish assemblage data to infer estuarine function. Aggregated FEAS scores enable comparisons across years, sites or catchments, offering insights into spatial variation in ecological value and functional importance. Together with traditional community metrics such as abundance and species richness, these tools provide a robust framework for evaluating ecological change, restoration targets and habitat value over time.

The Solent, a large temperate estuarine system in southern England, presents a valuable case study for investigating long‐term changes in nearshore fish assemblages. This study uses a 12‐year dataset (2007–2018) of seine net surveys conducted at 15 sites across four catchments to assess spatio‐temporal trends in fish abundance, species richness, functional guild composition and estuarine dependency (via FEAS). By applying FEAS metrics to historical community data, we can infer the estuarine functional value of different habitats and quantify variation in estuarine dependency. Importantly, this analysis establishes an ecological baseline for the Solent against which the current condition and functional state of the system can be assessed. This baseline supports ongoing restoration initiatives and provides a foundation for future monitoring and management recommendations in this highly urbanised estuary. Moreover, the approach developed here offers a transferable framework for assessing ecological function and change in other large estuarine systems where long‐term fish community data are available.

## 2 MATERIALS AND METHODS

### 2.1 Ethics statement

The care and use of fish complied with UK animal welfare laws and guidelines. All sampling was conducted under non‐invasive survey protocols approved by the Environment Agency and relevant Inshore Fisheries and Conservation Authorities. Fish were captured using seine nets, identified, counted and released alive at the capture site. No fish were harmed, held or killed for tissue sampling. Handling time was minimised, fish were held in aerated recovery buckets and all efforts were made to reduce stress and ensure animal welfare throughout the study.

### 2.2 Study location

**FIGURE 1:**
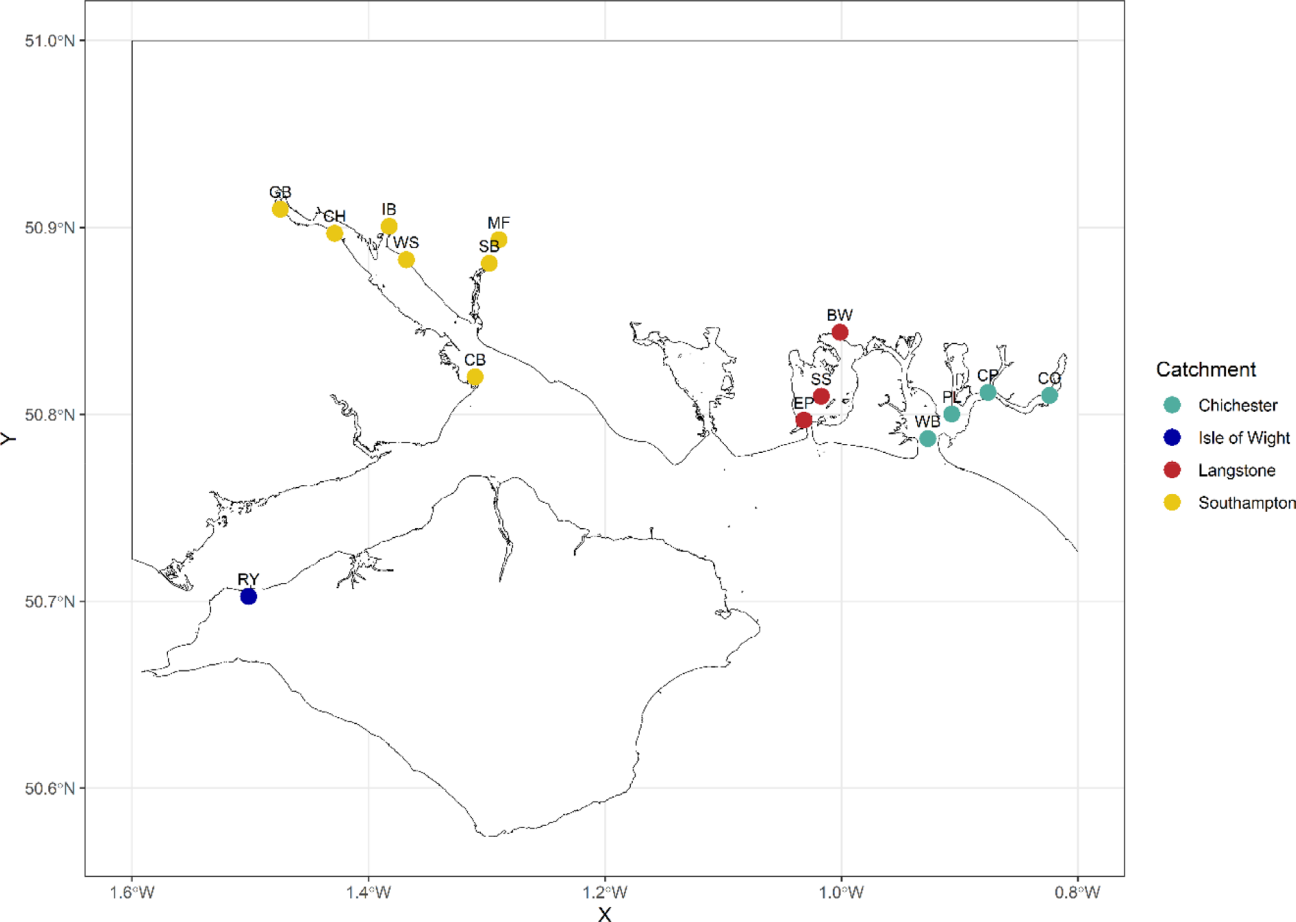
Survey sites are grouped by catchment and colour‐coded accordingly: green, Chichester Harbour; blue, Isle of Wight; red, Langstone Harbour; yellow, Southampton Water. Site names are labelled from left to right: RY, River Yar; BW, Bedhampton Wharf; CB, Calshot Beach; CH, Cracknore hard; CO, Copperas; CP, Cobnor Point; EP, Eastney Point; GB, Goatee beach; IB, Itchen Bridge; MF, Manor Farm Jetty; PL, Pilsey; SB, Swanwick Bend; SS, Sword Sands; WB, Winner Bank; WS, Weston Shore. Data represent the locations of biannual seine net surveys between 2007 and 2018.

The Solent, a strait between the north coast of the Isle of Wight and the south coast of mainland Great Britain, is a sediment‐dominated system comprising 12 distinct estuaries and harbours. The local topography, combined with the region's diurnal tidal fluctuation, creates a double high‐water phenomenon. The Solent's hydrology is characterised by a low‐energy, shallow, macro‐tidal system with a mean tidal range of 3.2 m (Iriarte & Purdie, 2004). The Solent has a variety of essential fish habitats including natural harbours, saltmarshes, seagrass, oyster reefs, kelp, other mixed macrophytes and rocky ledges (Watson, 2020). These habitats all play a key role in fish behaviour and movement. This study utilises 15 survey sites within four major catchments: Southampton Water, Langstone Harbour, the Isle of Wight and Chichester Harbour (Figure 1).

### 2.3 Survey methodology

Surveys were conducted at 15 sites across the Solent, covering multiple catchments with biannual monitoring in summer (June) and autumn (September–October) from 2007 to 2018. A total of 436 hauls were completed, 214 in summer and 222 in autumn. Sampling took place during daylight hours (6 a.m.–5 p.m.) at slack high or low tide, depending on site accessibility.

At each site, two replicate hauls were performed using a 43 × 4 m seine net with 6.5‐mm mesh in the centre panel and 14‐mm mesh in the wings. The net was deployed either from the shore or using a small tender, depending on access, and hauled ashore by a team of six to 10 people. Captured fish were placed immediately into aerated 40‐L buckets of seawater, identified to species level, and counted. Common species were processed and released on‐site; cryptic or unidentified individuals were photographed for later identification. All fish were placed in recovery buckets prior to being returned to the site.

### 2.4 Dataset generation and collation

Datasets were compiled from five organisations: The Environment Agency (EA), the Sussex Inshore Fisheries and Conservation Authority (SxIFCA), Langstone Harbour Board (LHB), the Southern Inshore Fisheries and Conservation Authority (SIFCA), and Chichester Harbour Conservancy (CHC). EA surveys originally included fyke and beam trawl methods designed to support Water Framework Directive (WFD) assessments. However, to ensure consistency across all data sources, only seine net data were used in this study because this was the common method employed by all organisations. Data were then organised by site, catchment, year, season and tidal state, providing broad spatial and temporal coverage of nearshore fish communities across the Solent.

Species names were updated using the World Register of Marine Species (WoRMS), and identifications were cross‐checked with FishBase (Froese & Pauly, 2019). *Sprratus sprattus* (Linnaeus, 1758), *Clupea harengus* (Linnaeus, 1758) and *Sardina pilchardus* (Walbaum, 1792), were grouped under the family Clupeidae due to difficulties identifying juvenile individuals to species level in the field. Their FEAS scores and guild classifications were based on mean values across the three species.

**FIGURE 2:**
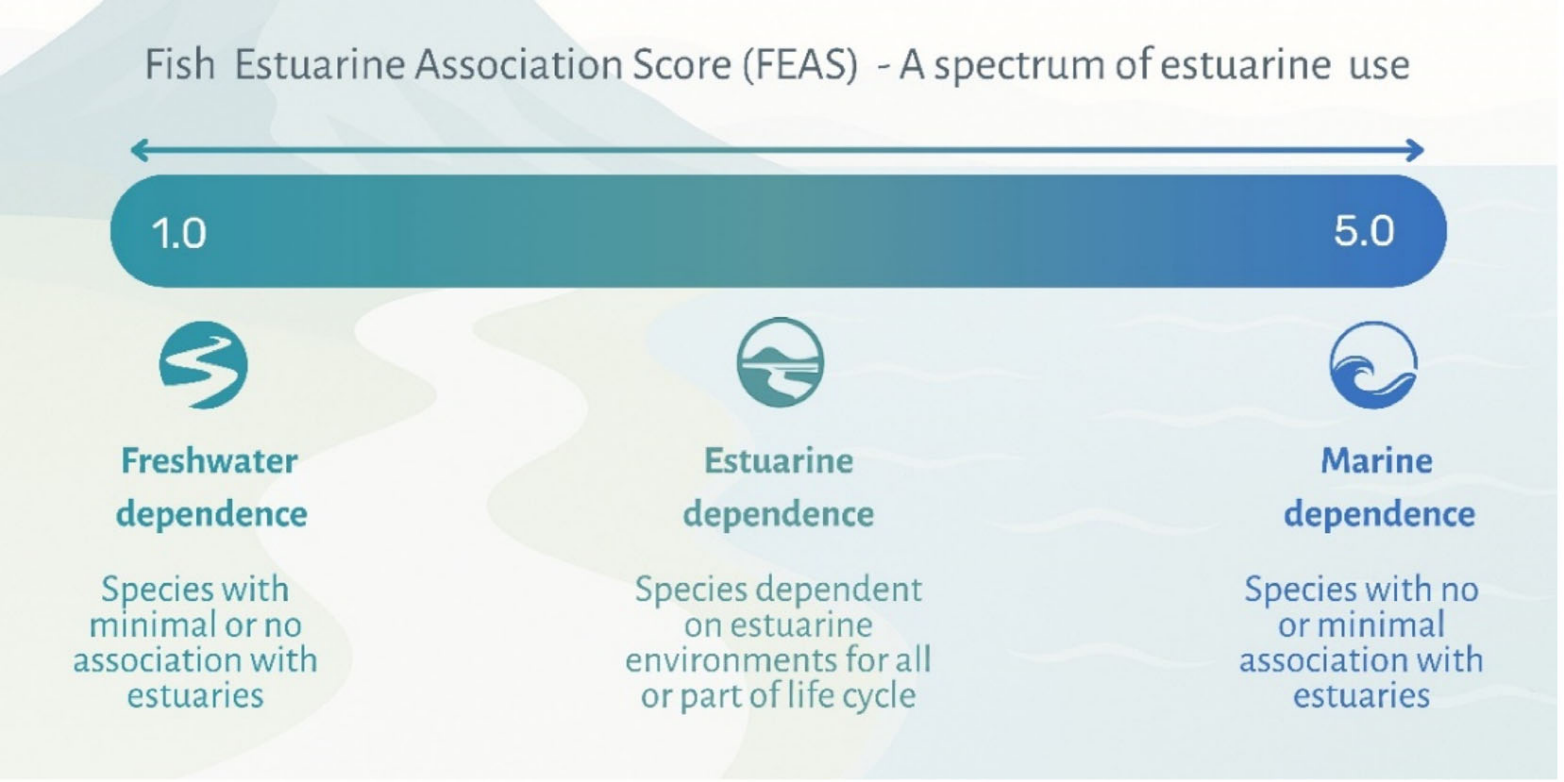
The spectrum of estuarine use based on FEAS scores from Harrison and Whitfield (2021), with scores of 1 being associated with freshwater species and scores of 5 associated with marine dependent species.

Each species was assigned to an Estuarine Use Functional Guild (EUFG) based on its typical frequency and purpose of estuarine use following the classification frameworks of Elliott et al. (2007) and Franco et al. (2008). EUFG categories included estuarine species (ES), marine migrants (MM), marine stragglers (MS), anadromous (AN), catadromous (CA) and freshwater species (FS). Fish Estuary Association Scores (FEAS) were assigned using the FEAS database, which contains estuarine association scores for some 6300 fish species (Harrison & Whitfield, 2021; available at https://doi.mba.ac.uk/data/1439). These species‐level scores range from 1.0 to 5.0 and quantify the degree of reliance on estuarine environments (with the full spectrum illustrated in Figure 2). Each species in our dataset was directly matched with its corresponding FEAS value in the database.

The final dataset includes site, catchment, year, season, tidal state, haul ID, species name, family, EUFG, FEAS and count. This formed the basis for assessing spatial and temporal variation in fish populations and community composition across the Solent. Table 1 details catchment, site name, years surveyed and tidal state.

**TABLE 1:** Associated catchment, site name, the total number of surveys conducted, latitude and longitude of each site, number of survey years and tidal state of each site.

Catchment	Latitude	Longitude	Site name	Tidal state	Survey count	Years surveyed
Chichester	50.8116	−0.8759	Cobnor point	High	3	2010–2012
Isle of Wight	50.7025	−1.5013	River Yar	High	3	2016–2018
Chichester	50.7870	−0.9268	Winner bank	Low	5	2010–2012, 2016, 2018
Langstone	50.7968	−1.0316	Eastney point	Low	6	2012–2017
Langstone	50.8097	−1.0169	Sword sands	Low	6	2012–2017
Langstone	50.8438	−1.0011	Bedhampton	High	6	2012–2017
Chichester	50.8001	−0.9065	Pilsey	Low	7	2010–2014, 2016, 2018
Chichester	50.8101	−0.8238	Copperas	High	8	2010–2014, 2016–2018
Southampton	50.9097	−1.4744	Goatee beach	High	9	2010–2018
Southampton	50.8199	−1.3097	Calshot	Low^a^	9	2010–2018
Southampton	50.8934	−1.2895	Manor farm jetty	Low	12	2007–2018
Southampton	50.8808	−1.2977	Swanwick bend	Low	12	2007–2018
Southampton	50.9005	−1.3823	Itchen bridge	High	12	2007–2018
Southampton	50.8827	−1.3678	Weston shore	High	12	2007–2018
Southampton	50.8968	−1.4285	Cracknore hard	High	12	2007–2018

^a^ Calshot was surveyed at high tide in 2010 and 2011.

### 2.5 Data analysis

To model temporal trends in fish abundance, a negative binomial generalised linear mixed model (GLMM) was applied to raw count data, with year as a fixed effect and season, haul and site as random effects. This approach accounted for overdispersion and was selected based on model fit. All variables were checked for normality and standardised prior to analysis. To assess variation in species composition across temporal and spatial factors, including year, season, tidal state, site and catchment, a permutational multivariate analysis of variance (PERMANOVA; Anderson, 2017) was conducted using a Bray–Curtis dissimilarity matrix on square root‐transformed data with 999 permutations. Where significant differences were detected, post hoc tests were used: the Kruskal–Wallis test for comparisons involving more than two groups and Mann–Whitney *U* tests for two‐group comparisons. Dunn's tests with Bonferroni‐adjusted *p* values were used for pairwise contrasts where appropriate. Non‐metric multidimensional scaling (NMDS) based on Bray–Curtis similarity matrices were used to visualise spatial and temporal patterns in fish community composition across season, tidal state, site and catchment. All analyses were conducted in R version 4.4.1 using the packages glmmTMB, dplyr, ggplot2, vegan, FSA and tidyr.

## 3 RESULTS

### 3.1 Diversity and functional guilds

Between June 2007 and September 2018, a total of 55 fish species and 141,917 individuals were recorded across 15 sites in the Solent. Species richness per site ranged from 10 to 33 (mean ± SD 18.5 ± 7.1, *n* = 15). Six dominant taxa, Clupeidae, *Atherina presbyter* Cuvier, 1829, *Dicentrarchus labrax* (Linnaeus, 1758), *Pomatoschistus microps* (Krøyer, 1838), *Pomatoschistus minutus* (Pallas, 1770) and *Chelon auratus* (Risso, 1810), accounted for 96% of all individuals. Among families, Atherinidae was the most abundant (28.3%), followed by Clupeidae (28.1%) and Moronidae (21.4%). Marine migrants (MM) dominated the dataset overall, comprising 82.4% of the total count. Estuarine residents (ES) accounted for 16.9%, with both guilds represented by the six most abundant species. In contrast, marine stragglers (MS), diadromous species (catadromous and anadromous) and freshwater stragglers collectively contributed less than 1%. Guild composition is summarised in Table S1. Several rarely encountered species including *Anguilla anguilla* (Linnaeus, 1758) and *Salmo trutta* Linnaeus, 1758 were recorded in low numbers. Full species counts are presented in Table S1. The mean Fish Estuary Association Score (E‐FEAS) was mean ± SD 3.95 ± 0.34 (*n* = 246), with the lowest score of 3.81 in 2007 and maximum of 4.07 in 2012.

### 3.2 Temporal patterns in abundance, richness and community composition

**FIGURE 3:**
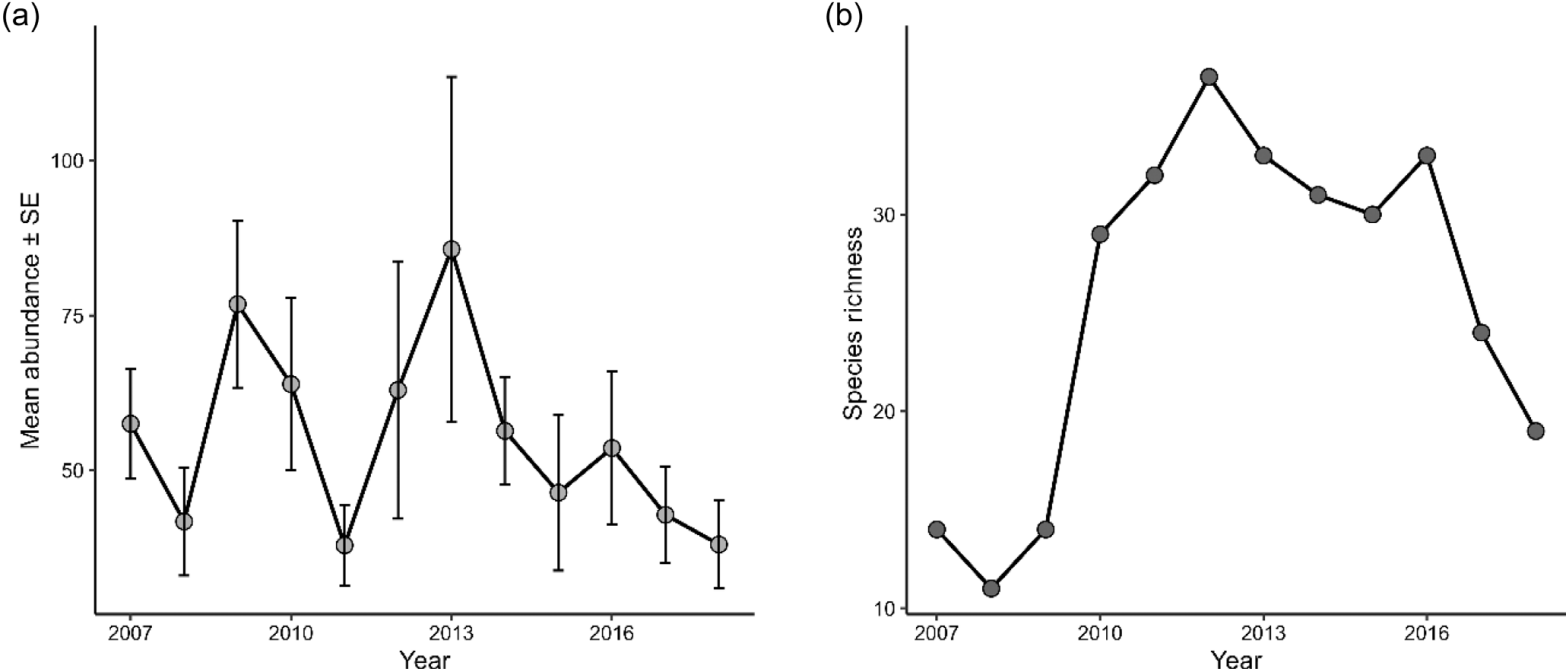
Temporal trends in fish community from 2007 to 2018 across all survey sites. (a) The average fish abundance per survey (mean ± SE) for each year. (b) Species richness, defined as the total number of taxa recorded annually. Note that the *y* axes in (a) and (b) differ.

Fish abundance fluctuated significantly over the study period, beginning at 7994 individuals in 2007, peaking at 24,167 in 2013 and declining to 6157 by 2018. Survey coverage varied across years, with five sites sampled in 2007–2009, 14 in 2012 and 2016, and 11 in 2018. The increase in abundance observed between 2010 and 2013 coincided with the expansion in site coverage. Species richness followed a broadly similar pattern, rising from 14 species in 2007 to 37 in 2012, then dropping to 19 by 2018 (Figure 3), although survey coverage did not decline at the same time.

**FIGURE 4:**
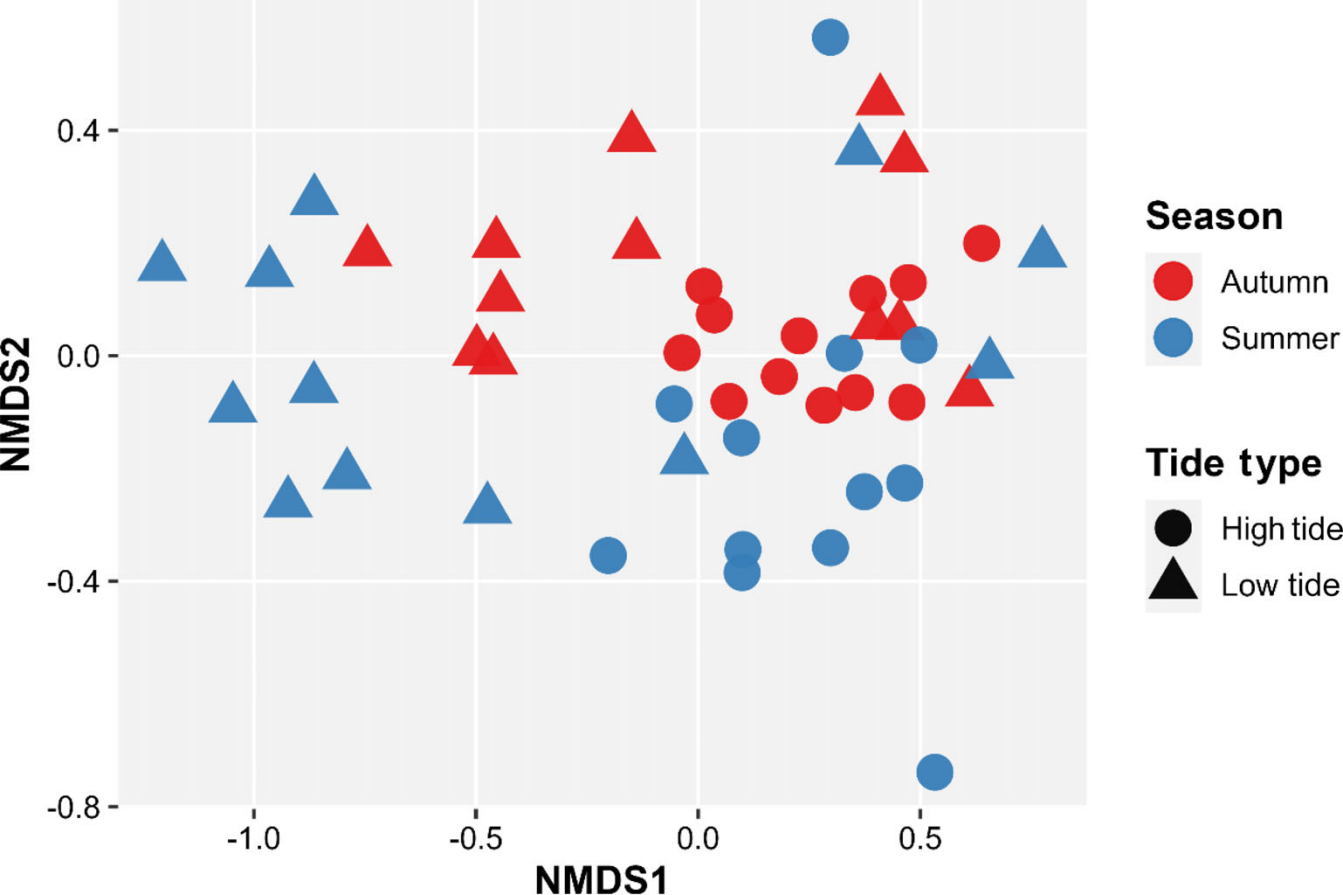
Non‐metric multidimensional scaling (NMDS) ordination plot showing seasonal variation in fish community composition across all sites and years, based on Bray–Curtis dissimilarity of square root‐transformed abundance data. Points represent survey year, coloured by season (red, autumn; blue, summer) and shaped by tide type (circle, high tide; triangle, low tide).

NMDS revealed temporal and tidal variation in fish community composition (stress = 0.148). Autumn and summer samples showed partial separation along the NMDS axes, indicating seasonal variation in assemblage structure. Tidal state also contributed to compositional differences, with high‐tide samples (circles) forming tighter clusters and low‐tide samples (triangles) more widely dispersed, suggesting greater variability in community structure at low tide (Figure 4).

A negative binomial generalised linear mixed model revealed a significant decline in fish abundance from 2007 to 2018, with a negative year coefficient (*β* = −0.098, *p* = 0.0114). PERMANOVA confirmed a significant effect of year on fish community composition (*df* = 11, F = 1.94, *p* = 0.002). Pairwise comparisons revealed that 2008 differed significantly from multiple years, including 2010 (*p* = 0.026), 2011 (*p* = 0.019), 2012 (*p* = 0.029), 2013 (*p* = 0.038), 2014 (*p* = 0.033) and 2015 (*p* = 0.022). Additional significant differences were found between 2007 and both 2012 (*p* = 0.031) and 2015 (*p* = 0.036), as well as between 2010 and 2018 (*p* = 0.027), and between 2012 and 2018 (*p* = 0.021). Most other year‐to‐year comparisons were not significant.

**FIGURE 5:**
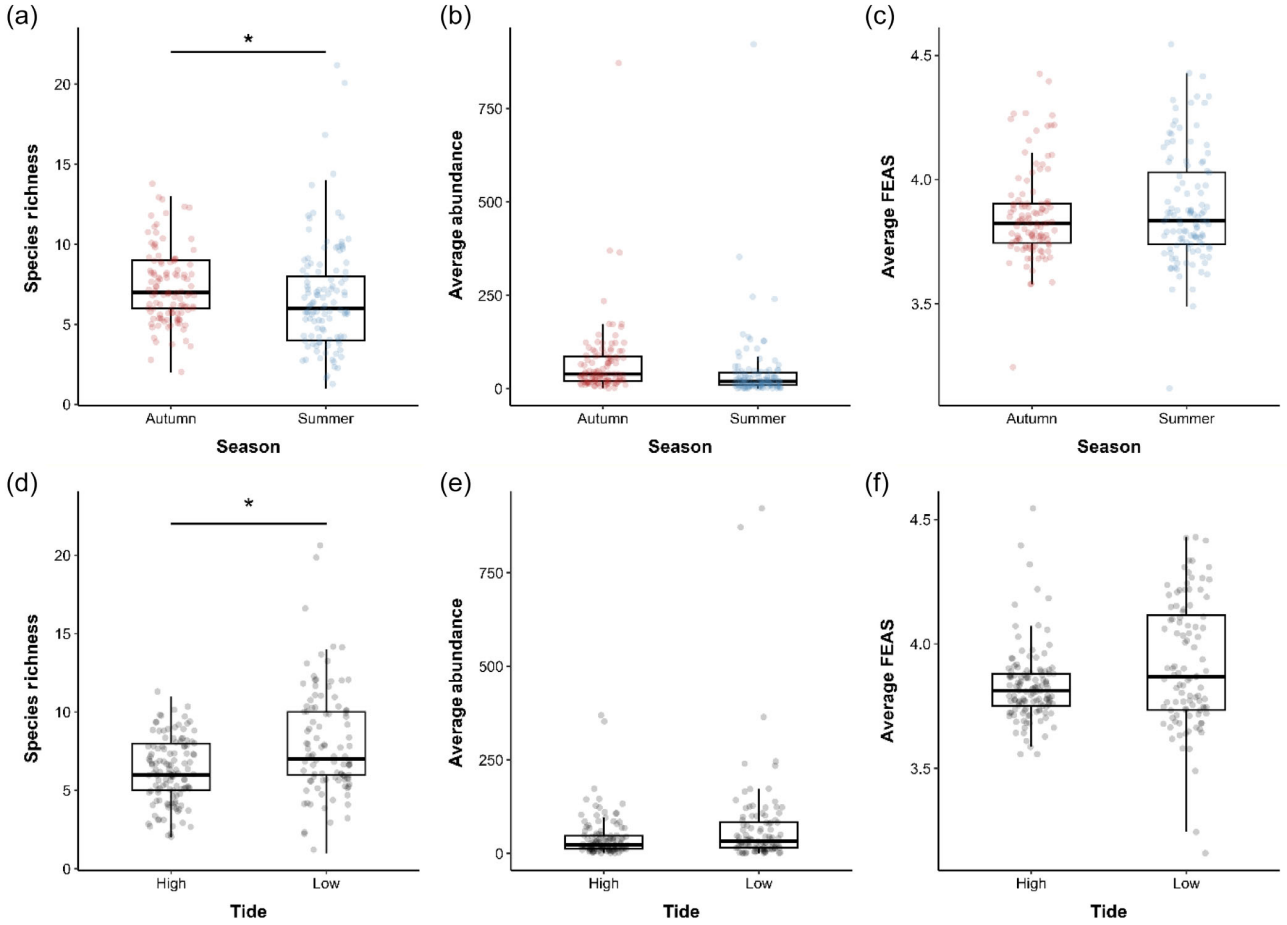
Box plots comparing fish community metrics across season (autumn vs. summer; a–c) and tide (high vs. low; d–f). (a, d) Species richness per survey. (b, e) Average fish abundance (mean number of individuals per survey). (c, f) Average Fish Estuarine Association Scores (FEAS), where lower values (closer to 3) indicate a stronger estuarine association. Asterisks (*) indicate statistically significant differences (*p* < 0.05). *y*‐axes differ between panels.

Season also significantly influenced fish community composition (PERMANOVA: *df* = 1, F = 8.09, *p* = 0.001), with a significant difference between autumn and summer communities (*R* ^2^ = 0.120, *F* = 6.28, *p* = 0.001). Species richness was significantly higher in autumn compared to summer (mean difference = −0.94, *t* = −3.43, *df* = 101, *p* = 0.0009), although no significant seasonal differences were detected for average abundance (*p* = 0.123) or FEAS scores (*p* = 0.327). Tidal state also had a significant effect on community composition (PERMANOVA: *df* = 1, *F* = 3.86, *R* ^2^ = 0.057, *p* = 0.001). Species richness was significantly higher at low tide compared to high tide (*W* = 4178, *p* < 0.001), but no significant differences were observed for abundance (*p* = 0.072) or FEAS scores (*p* = 0.059) (Figure 5).

### 3.3 Spatial patterns in abundance, richness and community composition

**FIGURE 6:**
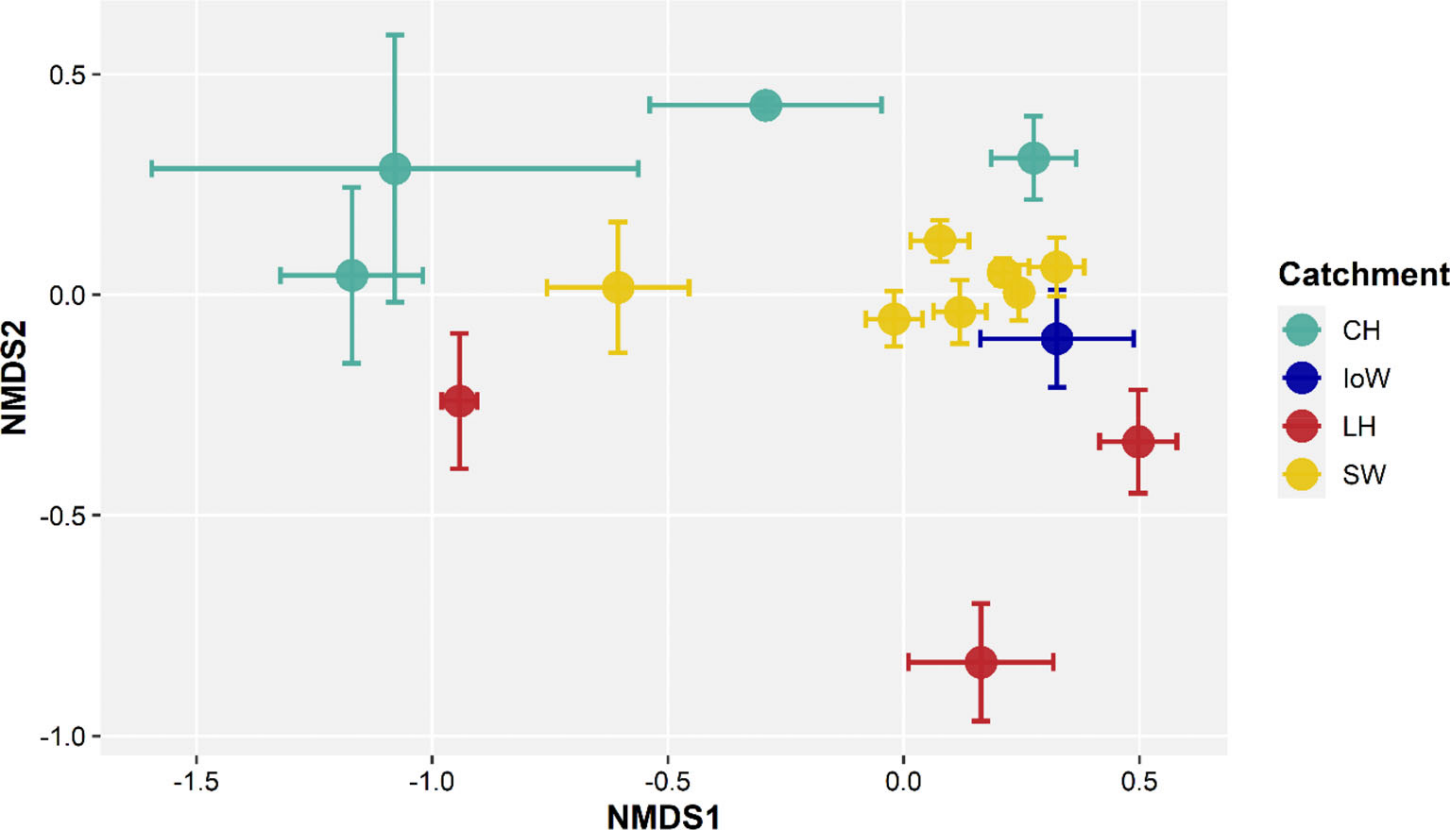
Non‐metric multidimensional scaling (NMDS) plot showing site centroids of fish community composition across catchments from 2007 to 2018. Points represent median NMDS1 and NMDS2 values with error bars. Colours indicate catchments. NMDS stress = 0.201. CH, Chichester Harbour; IoW, Isle of Wight; LH, Langstone Harbour; SW, Southampton Water.

Fish community composition showed clear spatial variation across the Solent over the 12‐year study period. PERMANOVA analysis using Bray–Curtis dissimilarity revealed a significant effect of year (*df* = 11, *F* = 7.61, *p* = 0.002), catchment (*df* = 3, *F* = 8.25, *p* = 0.001) and site (*df* = 14, *F* = 6.93, *p* = 0.001), with NMDS ordination (stress = 0.201) showing visible separation in community composition among catchments (Figure 6).

At the catchment scale, Southampton Water (SW) recorded the highest fish abundance (88,277 individuals) and species richness (45 species), with contributions from six estuarine use functional guilds. Chichester Harbour (CH) and Langstone Harbour (LH) also supported notable diversity, with four and three guilds respectively. The Isle of Wight (IW) recorded the lowest abundance (1624 individuals) and richness (11 species), with only two guilds represented. CH and SW were the only catchments to support both anadromous and catadromous species.

**FIGURE 7:**
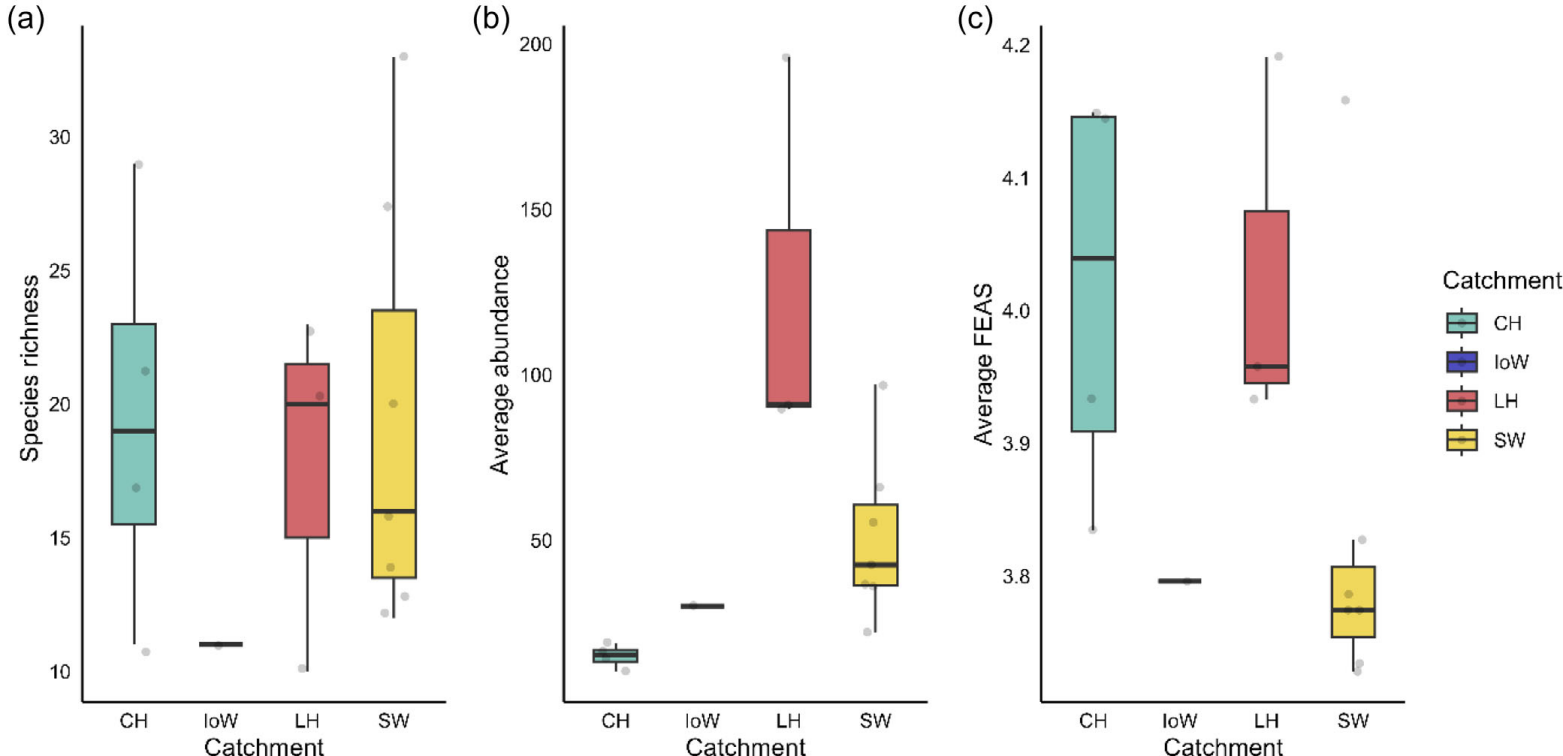
(a) Box plots of species richness recorded in each catchment. (b) Average fish abundance across all years for the four catchments. (c) Average Fish Estuarine Association Scores (FEAS), where FEAS values closer to three indicate a stronger association with estuarine habitats. CH, Chichester Harbour (green); IoW, Isle of Wight (blue); LH, Langstone Harbour (red); SW, Southampton Water (yellow).

Species richness did not differ significantly between catchments (*χ* ^2^ = 7.17, *df* = 3, *p* = 0.666), but average abundance (*χ* ^2^ = 170.52, *df* = 3, *p* < 0.001) and FEAS scores (*χ* ^2^ = 20.48, *df* = 3, *p* < 0.001) did. (Figure 7). Post hoc Dunn's tests showed that Chichester Harbour (CH) had significantly lower fish abundance than both Langstone Harbour (LH; *p* = 0.0007) and Southampton Water (SW; *p* = 0.007). FEAS scores were significantly higher in CH and LH compared to SW (*p* = 0.0025 and *p* = 0.004, respectively), while the Isle of Wight did not differ significantly from any other catchment.

At the site level, Eastney Point (EP) had the highest fish abundance (27,082 individuals), and Winner Bank (WB) the lowest (901). Calshot recorded the greatest species richness (33 species) and five guilds, while River Yar (RY) supported only two. Post hoc tests showed significant variation in species richness between sites (*χ* ^2^ = 42.56, *df* = 14, *p* < 0.001), with EP and Pilsey (PL) significantly higher than Weston Shore (WS) (*p* = 0.049 and *p* = 0.039, respectively). Fish abundance also differed significantly across sites (*χ* ^2^ = 63.70, *df* = 14, *p* < 0.001). Swanwick Bend (SB) had significantly higher abundance than Copperas (CO) (*p* = 0.002), PL (*p* = 0.003), WB (*p* = 0.001) and WS (*p* = 0.001), while Itchen Bridge (IB) was also higher than CO (*p* = 0.021), PL (*p* = 0.028), WB (*p* = 0.008) and WS (*p* = 0.011). FEAS scores differed significantly across sites (*χ* ^2^ = 80, *df* = 14, *p* < 0.001), with EP higher than IB (*p* = 0.015), MF (*p* = 0.001) and SB (*p* = 0.001), and PL higher than CH (*p* = 0.032), GB (*p* = 0.025), IB (*p* = 0.006), MF (*p* < 0.001) and SB (*p* < 0.001).

## 4 DISCUSSION

### 4.1 Diversity and functional guilds

This study examined fish community composition, diversity and abundance across 15 sites in the Solent over a 12‐year period. A total of 55 species were recorded, highlighting a diversity level comparable to other temperate, urbanised estuaries in the UK and Europe (Elliott et al., 2007; Gibson et al., 2024). Although some regions, such as the Severn Estuary and Bristol Channel, can support over 110 species, Franco et al. (2008) reported an average of 53 ± 20 species across 38 European estuaries, suggesting that the Solent's diversity is consistent with many degraded estuarine and coastal systems. Six species dominated the community, accounting for 96% of all individuals, and were exclusively from the marine migrant (MM) and estuarine resident (ES) functional guilds. This pattern aligns with previous findings that estuarine fish communities are typically composed of a few abundant, persistent species and support lower species richness than adjacent marine systems (Magurran & Henderson, 2003; Martino & Able, 2003). The use of functional guilds to classify species by ecological role, behaviour and habitat use has proven effective in understanding community dynamics and environmental responses (Elliott et al., 2007; Garrison & Link, 2000; Lai et al., 2024; Mathieson et al., 2000; Silva et al., 2022). In the Solent, the dominance of marine migrants (82.3%) underscores strong ecological connectivity between estuarine and coastal waters (Ferreira et al., 2019), consistent with patterns in other Atlantic estuaries where marine migrants, catadromous, and estuarine residents dominate assemblages (Elliott et al., 2007; Mathieson et al., 2000; Selleslagh & Amara, 2008). The ecological dominance of marine migrants in the Solent also reflects its role as a key transitional zone between coastal and estuarine environments. For example, *D. labrax* larval supply to the Solent originates predominantly from spawning areas in the Channel (Graham et al., 2023). Particle‐tracking models have demonstrated that, despite juvenile sea bass exhibiting strong site fidelity, dispersal among adjacent estuaries (often less than 50 km apart) creates a key link between local nursery habitats and offshore populations (Graham et al., 2023). Similarly, *Atherina presbyter* has been known to have a spawning location in coastal algae beds near the entrance to Southampton Water before moving into deeper offshore waters of the Solent (Tubbs, 1999). This ontogenetic movement indicates that the estuary supports early life stages and facilitates the mixing of populations that contribute to Channel fish stocks.

The three most abundant taxa, *A. presbyter* (28.3%), Clupeidae (28.1%) and *D. labrax* (21.4%), illustrate the functional diversity of the Solent as a dynamic coastal ecosystem. *A. presbyter* is tolerant of variable salinities and low oxygen conditions (Almeida et al., 2024; Lima et al., 2024), which likely explains its widespread distribution despite anthropogenic pressures. Members of the Clupeidae family, including *Clupea harengus*, *Sprattus sprattus* and *Sardina pilchardus*, play a critical role in trophic energy transfer as both predators and prey (Garrison & Link, 2000; Peck et al., 2021). For instance, juvenile *C. harengus* and *S. sprattus* display opportunistic feeding on copepods and benthic invertebrate larvae, thereby facilitating energy flow across trophic levels and between benthic and pelagic habitats (Maathuis et al., 2024). As a highly mobile species, *D. labrax* links coastal and offshore environments through distinct life‐stage movements. Telemetry studies show juveniles often remain in nursery areas for up to 5 years before moving offshore, while adults migrate seasonally between productive inshore feeding zones and deeper offshore spawning and overwintering habitats along the southern UK coastline (Pawson et al., 1987, 2007; Pickett et al., 2004). In addition to its ecological role, *D. labrax* is of high recreational and economic value (Dawson et al., 2024; Fernández Sánchez et al., 2021; Tidbury et al., 2021; Vandeputte et al., 2019), a status reflected in the designation of extensive bass nursery areas protected by bylaws aimed at conserving juvenile populations (MMO, 2023). These nursery habitats are vital for providing shelter and foraging opportunities, and for enabling migration to offshore environments, further strengthening the broader ecological connectivity that supports commercial fisheries (Freeman et al., 2024; James et al., 2019; Pawson et al., 2007; Stamp et al., 2022). While the essential role of estuarine nursery habitats in sustaining adult fish stocks is widely acknowledged, the quantitative contribution of juveniles to coastal populations remains poorly understood (Gillanders, 2005; Vasconcelos et al., 2011). This functional connectivity is particularly critical for commercially important species because deteriorating estuarine conditions can reduce juvenile survival, ultimately leading to declines in adult populations. This, in turn, may disrupt trophic flow, ecosystem resilience and the sustainability of regional fisheries (Ikpewe et al., 2021; Reis‐Santos et al., 2013; Simpson et al., 2011; Swadling et al., 2024; Thornborrow et al., 2024).

### 4.2 Temporal changes in fish abundance and community composition

Temporal analyses revealed a significant decline in fish abundance in the Solent from 2007 to 2018, consistent with global patterns of declining fish populations in degraded estuarine habitats (Belarmino et al., 2021; Whitfield, 2017; Whitfield et al., 2018). Estuaries are among the most degraded habitats globally (Jung et al., 2024), facing sustained anthropogenic pressures such as habitat modification, pollution and climate change (Kennish, 2022, 2023; Kennish et al., 2024). These stressors contribute to the decline of estuary‐associated marine fishes observed across regions (Whitfield, 2021), and the patterns observed in the Solent mirror broader biodiversity loss in these critical coastal systems (Lepage et al., 2022; O'Leary et al., 2021; Stamp et al., 2022).

Our results also showed significant temporal variability in species richness and community composition between summer and autumn. Seasonal differences in community structure align with previous studies that report consistent seasonal shifts in estuarine fish assemblages (Chen et al., 2022; Claridge et al., 2009; Hagan & Able, 2003). For instance, Lee et al. (2014) found strong seasonal contrasts using both gillnetting and environmental DNA (eDNA) in estuarine environments, while Koutrakis et al. (2000) reported higher richness and abundance during warmer months in the Rihios system. These seasonal dynamics are likely driven by factors such as temperature, recruitment pulses (Mir‐Arguimbau et al., 2022) and prey availability (Ouellet et al., 2025). Although temperature was not directly measured, warmer sea surface temperature in autumn (Cornes et al., 2023) are known to elevate metabolic demand (Volkoff & Rønnestad, 2020), enhance recruitment (Lourenço et al., 2023) and shift prey communities (Schoenebeck et al., 2024), all of which can restructure fish assemblages (Colombano et al., 2022; Guo et al., 2022; Lai et al., 2024). In species such as *D. labrax*, recruitment success and larval supply are highly temperature‐dependent (Cabral et al., 2021), contributing to seasonal changes in functional group abundance and trophic interactions (Elliott et al., 2007). In addition, Vendel et al. (2003) observed seasonal shifts in fish abundance, with fewer fishes being captured during winter and part of spring. Furthermore, tidal state also influenced community composition, with species richness being significantly higher at low tide. Other studies, such as Gaelzer and Zalmon (2008) and Morrison et al. (2002), also noted increased richness and abundance at low tide, reinforcing the role of tidal exposure in structuring fish communities. Gibson et al. (1996) documented intertidal movements associated with rising tides, while Teather et al. (2012) reported minimal differences between flood and ebb tides, highlighting variability based on local context. In the Solent, the lack of significant differences in abundance and FEAS scores between high and low tides suggests that although species identities shift, overall biomass and functional roles remain relatively stable. Overall, these results emphasise the importance of considering temporal shifts in abundance, richness and functional guild composition across multiple scales, annually, seasonally and daily, when assessing estuarine fish populations.

### 4.3 Spatial differences between catchment and sites

Fish community composition, abundance, and estuarine dependency (as measured by FEAS) varied significantly across both catchments and individual sites within the Solent. These patterns are consistent with previous studies showing that estuarine fish assemblages are shaped by both broad‐scale gradients and fine‐scale habitat features (Elliott et al., 2007; Harrison & Whitfield, 2006; Maciel et al., 2024; Martinho et al., 2012). For example, Goodridge Gaines et al. (2022) found that spatial context, particularly proximity to estuary mouths, intertidal flats and vegetated habitats, was more predictive of fish abundance and diversity than habitat condition alone. Among the four catchments, Southampton Water supported the highest fish abundance and species richness, and the widest range of estuarine use functional guilds. This likely reflects a combination of factors; the catchment encompasses a variety of habitats (e.g. mudflats, saltmarsh, shallow subtidal zones and artificial structures), includes the highest number of survey sites and had the longest sampling duration. Calshot, within this catchment, supported the greatest species richness and five guilds, suggesting that local habitat diversity and ecological connectivity can enhance the availability of niches for different life‐history strategies, supporting a more functionally balanced assemblage (Sreekanth et al., 2020). In contrast, nearby sites such as Cracknore Hard supported fewer guilds, highlighting substantial within‐catchment variation. Such patterns emphasise how nearshore and juvenile fish respond to local features such as cover, food availability and hydrodynamic conditions.

Despite its urbanisation, Southampton Water had significantly lower FEAS scores compared to Chichester and Langstone Harbours, indicating greater dominance by estuarine‐dependent species, those with FEAS values closer to 3.0. These taxa are functionally linked to estuarine environments for critical stages of their life cycles and are more vulnerable to habitat degradation (Harrison & Whitfield, 2021). In contrast, Chichester Harbour, while less urbanised and containing fewer surveyed sites, supported high species richness and multiple estuarine‐use guilds. Pilsey, located near extensive saltmarsh and intertidal flats, had the second‐highest species richness of all sites despite reduced sampling coverage, underscoring the importance of natural habitat quality and connectivity (Mosman et al., 2024; Van Lier et al., 2018). The presence of dedicated management bodies such as the Chichester Harbour Conservancy (CHC, 2024) and strong spatial protections from IFCA byelaws (IFCA, 2024) may further contribute to the biodiversity observed. In contrast, sites like Winner Bank, more exposed and further from sheltered nursery areas, had the lowest average abundance. This reflects broader findings that proximity to sheltered, productive habitats such as creeks and vegetated flats drives community structure and function in estuarine systems (Bulleri & Chapman, 2010; Burt & Bartholomew, 2019; Clauzel & Godet, 2020; Green et al., 2021; Nagelkerken et al., 2015).

At the site level, spatial heterogeneity was even more distinct. Calshots' high richness and guild diversity likely reflect its proximity to seagrass beds and relatively sheltered location, features known to enhance nursery value and fish diversity (Janes et al., 2021; McHenry et al., 2021; Unsworth et al., 2019). In contrast, sites such as Eastney Point recorded the highest abundance but had higher FEAS scores, indicating assemblages dominated by marine stragglers or species with weaker estuarine reliance. This suggests that high abundance does not necessarily equate to high functional estuarine importance (Teichert et al., 2017). Swanwick Bend and Manor Farm Jetty, both of which recorded FEAS scores closer to 3.0, were instead characterised by stronger use from estuarine‐dependent species. These intra‐catchment differences reinforce the idea that ecological value cannot be assumed to be uniform within estuarine systems, even over small spatial scales (Davis et al., 2020; Pessanha et al., 2021). Site‐specific habitat features such as sediment type, structural complexity and exposure strongly influence both species composition and functional roles (Kuang et al., 2021).

Notwithstanding the relatively compact scale of the Solent, the spatial variation in abundance, richness and FEAS across its sites highlights the complexity of estuarine nursery function. Sites like Pilsey and Calshot support a wider range of guilds or higher estuarine dependency, making them especially important in terms of functional biodiversity. These findings are consistent with other studies that show the strongest nursery function often occurs where natural habitats are well‐connected and minimally disturbed (Gittman et al., 2016; James et al., 2019; Sagerman et al., 2019). Conversely, degraded or fragmented habitats may still support fish, but with reduced diversity and diminished estuarine functional value (Vasconcelos et al., 2011). Incorporating FEAS alongside traditional metrics such as abundance and species richness offers a powerful framework for interpreting spatial variability in estuarine function (Harrison & Whitfield, 2021). While richness and abundance reflect diversity and productivity, FEAS captures the degree to which sites are relied on by estuarine‐dependent species. By combining FEAS with functional guild analysis, this study provides a multidimensional perspective on estuarine use, enhancing our ability to evaluate habitat quality and ecological value across space using historical data.

### 4.4 Implications and recommendations for restoration and management

Effective estuarine management depends on a clear understanding of fish community structure, function and habitat use. By integrating species richness, abundance, functional guilds (EUFG) and estuarine dependency (FEAS), this study provides a detailed assessment of ecological variation across the Solent. Together, these metrics offer a useful framework for identifying sites that support key life stages of estuarine‐associated fish and for interpreting how fish communities respond to spatial and temporal change. The dataset provides an important ecological baseline at a time when large‐scale marine restoration is already underway in the region (BMF, 2024). Capturing pre‐restoration conditions is crucial to evaluating future change, helping to distinguish genuine ecological recovery from natural variability.

Measuring the success of restoration projects is often challenging, particularly in dynamic systems like estuaries. Projects are frequently constrained by short funding cycles, limited spatial coverage or missing baseline data (Basconi et al., 2020; Bayraktarov et al., 2016; Cooke et al., 2019; Danovaro et al., 2021). These constraints are compounded in systems where long‐term ecological change has already occurred, making it difficult to define meaningful reference conditions (Alleway et al., 2023; Pauly, 1995). Without sustained monitoring across multiple sites, there is a risk of attributing natural fluctuations to restoration efforts. Recognising and accounting for this spatial and temporal complexity is therefore essential for setting realistic goals and for interpreting change with confidence (England et al., 2021; Schulz et al., 2020).

While seine nets remain a practical method for monitoring nearshore fish, they can be limited in complex or vegetated habitats and are labour‐intensive (NECR 271, 2020; Bayley & Herendeen, 2000). Looking ahead, incorporating complementary approaches such as baited remote underwater videos, fyke nets, standard monitoring units for the recruitment of fishes, eDNA and drop‐down cameras can improve species detection, habitat coverage and overall survey resolution (DiBattista et al., 2022; French et al., 2021). These methods, especially when linked to environmental variables like temperature, salinity or oxygen, enhance our understanding of what drives fish distribution and habitat use. However, historical data often lack these contextual details, particularly in multi‐agency datasets. In this context, applying retrospective functional metrics such as FEAS and estuarine‐use guilds offers a powerful way to infer ecological function and build a meaningful baseline from what data are available. This approach helps overcome the limitations of older survey methods and provides a structured basis for guiding conservation priorities, restoration planning and management decisions.

The results also support a more targeted approach to habitat management. Sites where fish assemblages are dominated by species with FEAS scores close to 3.0 are likely to have the greatest estuarine functional importance. These are the species that depend on estuaries for feeding, shelter and growth, and are most vulnerable to degradation (Harrison & Whitfield, 2021). Prioritising such sites for conservation and restoration will help safeguard nursery function and maintain fish populations over the long term. Conversely, sites with higher FEAS scores, typically dominated by more transient marine species, may be less functionally dependent on estuarine conditions and could require less intensive management. Incorporating FEAS and functional guild data into restoration planning also offers a way to identify ecologically valuable areas that might not be apparent from diversity or abundance data alone. Based on the key patterns observed in this study, several practical recommendations for estuarine monitoring design are summarised in Table 2.

**TABLE 2:** Priority monitoring recommendations for temperate estuarine fish assemblages based on this study's findings.

Priority	Recommendation	Rationale	Application/benefit
1	Maintain long‐term, multi‐site monitoring across all catchments	Captures spatial variability; essential for detecting real change vs. natural fluctuations	Supports site‐specific restoration planning and reduces risk of false inferences
2	Prioritise autumn sampling	Higher richness and functional diversity in autumn makes it most efficient for detecting estuarine‐associated species	If only one season is feasible, choose autumn. Increases detection of key nursery species, improving habitat valuation
3	Include both high and low tide sampling	Tidal state significantly influenced community composition and richness	Enhances understanding of habitat use across tidal cycles
4	Ensure seasonal replication (at least summer and autumn)	Community composition shifts seasonally; some functional groups appear only in one season	Captures full seasonal turnover in community and functional structure; important if assessing change over time
5	Balance catchment coverage and effort	Uneven sampling may bias comparisons and reduce representativeness	Ensures fair comparison and prioritisation across management units

*Note*: Recommendations are ranked by importance under typical logistical constraints (e.g. time, funding, access) and reflect key patterns in spatial and temporal variability in fish community structure, richness, abundance and estuarine dependency. This framework supports the design of efficient, evidence‐based monitoring strategies to inform restoration and management in other temperate estuarine systems.

As highlighted in Table 2, designing effective monitoring frameworks requires accounting for spatial heterogeneity and functional connectivity across catchments. Spatial variation within the Solent further highlights the importance of connectivity. Estuarine‐dependent species rely on a mosaic of linked habitats, from intertidal flats to subtidal zones and nearshore reefs to complete their life cycles (Graham et al., 2023; Nagelkerken et al., 2015). Maintaining access to these habitats, and ensuring that movement pathways remain intact, should be a key focus of any future restoration efforts. Sites that support multiple functional guilds may also offer greater ecological resilience, helping buffer fish communities against disturbance or environmental change (Sreekanth et al., 2020).

Finally, this study recorded a decline in fish abundance and changes in community composition between 2007 and 2018. These findings point to wider ecological changes underway in the Solent and underline the urgency of reversing habitat degradation. Establishing clear ecological baselines is vital not only to set realistic restoration goals, but also to ensure that any observed recovery can be confidently linked to management interventions. Long‐term monitoring, combined with functional assessments like FEAS, offers a practical way to track restoration outcomes and support more adaptive, evidence‐based management of estuarine systems.

## AUTHOR CONTRIBUTIONS

Zoe Morrall: Conceptualisation (lead), methodology (lead), investigation (lead), formal analysis (lead), data curation (lead), visualisation (lead), writing – original draft (lead), writing – review and editing (equal). George P. Balchin: Investigation (equal), writing – review and editing (equal). Jenifer Lewis: Investigation (equal), writing – review and editing (equal). Dominic Longley: Investigation (equal), writing – review and editing (equal). Nick Rogers: Investigation (equal), writing – review and editing (equal). Ian Hendy: Writing – review and editing (equal). Gordon Watson: Writing – review and editing (equal). Joanne Preston: Funding acquisition (lead), writing – review and editing (equal).

## FUNDING INFORMATION

This research was supported by the Solent Seascape Project, funded through a 5‐year grant from the Endangered Landscapes & Seascapes Programme, managed by the Cambridge Conservation Initiative and funded by Arcadia, and by the grant‐making charity East Head Impact.

## Supporting information


**TABLE S1.** Summary of fish species recorded in nearshore surveys of the Solent estuarine system (2007–2018), including common and scientific names, total counts, relative abundance (%), estuarine use functional guild (EUFG) and Fish Estuarine Association Scores (FEAS). FEAS values are presented as means ± standard error. Taxa are listed in descending order of total count. Species identifications were updated using the World Register of Marine Species and FishBase. FEAS and EUFG classifications follow Harrison and Whitfield (2021) and Elliott et al. (2007).
