# Supplementary material for: Spatio‐temporal assessment of nearshore fish communities in a temperate estuary using functional and community metrics for restoration and management
Source: J Fish Biol. 2025 Aug 7;107(5):1729–44. doi: 10.1111/jfb.70171 (PMC12710854; doi:10.1111/jfb.70171)
Supplement: Supplementary file 1 — TABLE S1. Summary of fish species recorded in nearshore surveys of the Solent estuarine system (2007–2018), including common and scientific names, total counts, relative abundance (%), estuarine use functional guild (EUFG) and Fish Estuarine Association Scores (FEAS). FEAS values are presented as means ± standard error. Taxa are listed in descending order of total count. Species identifications were updated using the World Register of Marine Species and FishBase. FEAS and EUFG classifications follow Harrison and Whitfield (2021) and Elliott et al. (2007). [file JFB-107-1729-s001.docx]

Table S1: Summary of fish species recorded in nearshore surveys of the Solent estuarine system (2007–2018), including common and scientific names, total counts, relative abundance (%), estuarine use functional guild (EUFG), and Fish Estuarine Association Scores (FEAS). FEAS values are presented as means ± standard error. Taxa are listed in descending order of total count. Species identifications were updated using WoRMS and FishBase. FEAS and EUFG classifications follow Harrison & Whitfield (2021) and Elliott et al. (2007).

| Common Name | Latin Name |  | Total Count | % | EUFG | FEAS |  |
| --- | --- | --- | --- | --- | --- | --- | --- |
| Clupeidae | *Clupeidae* |  | 39401 | 29.83 | MM |  |  |
| Sand smelt | *Atherina presbyter* |  | 38302 | 29.00 | MM | 4.09 ± 0.06 |  |
| Bass | *Dicentrarchus labrax* |  | 28646 | 21.69 | MM | 3.99 ± 0.05 |  |
| Common goby | *Pomatoschistus microps* |  | 10764 | 8.15 | ES | 3.16 ± 0.09 |  |
| Sand goby | *Pomatoschistus minutus* |  | 7823 | 5.92 | ES | 3.33 ± 0.10 |  |
| Golden grey mullet | *Chelon auratus* |  | 2021 | 1.53 | MM | 3.98 ± 0.02 |  |
| Thick lipped grey mullet | *Chelon labrosus* |  | 1088 | 0.82 | MM | 4.02 ± 0.04 |  |
| Grey mullet sp. | *Mugilidae* |  | 841 | 0.64 | MM |  |  |
| Thin lipped grey mullet | *Liza ramada* |  | 663 | 0.50 | MM | 3.52 ± 0.09 |  |
| Lesser sandeel | *Ammodytes tobianus* |  | 577 | 0.44 | MM | 3.90 ± 0.18 |  |
| Black goby | *Gobius niger* |  | 257 | 0.19 | ES | 3.30 ± 0.13 |  |
| Corkwing wrasse | *Symphodus melops* |  | 246 | 0.19 | MS | 4.50 ± 0.20 |  |
| Black Seabream | *Spondyliosoma cantharus* |  | 200 | 0.15 | MS | 4.67 ± 0.10 |  |
| European flounder | *Platichthys flesus* |  | 169 | 0.13 | MM | 3.56 ± 0.10 |  |
| Ballan wrasse | *Labrus bergylta* |  | 160 | 0.12 | MS | 5.00 ± 0.00 |  |
| Shanny | *Lipophrys pholis* |  | 116 | 0.09 | MS | 5.00 ± 0.00 |  |
| Pollock | *Pollachius pollachius* |  | 102 | 0.08 | MM | 4.38 ± 0.14 |  |
| Greater Sand Eel | *Hyperoplus lanceolatus* |  | 96 | 0.07 | MS | 4.54 ± 0.18 |  |
| European Plaice | *Pleuronectes platessa* |  | 73 | 0.06 | MM | 4.06 ±0.05 |  |
| 15 Spined Stickleback | *Spinachia spinachia* |  | 72 | 0.05 | ES | 3.57 ± 0.21 |  |
| Garfish | *Belone belone* |  | 61 | 0.05 | MM | 4.38 ± 0.11 |  |
| Rock goby | *Gobius paganellus* |  | 59 | 0.04 | ES | 3.45 ± 0.18 |  |
| Long-spined sea scorpion | *Taurulus bubalis* |  | 58 | 0.04 | MS | 4.70 ± 0.15 |  |
| 2 Spot Goby | *Gobiusculus flavescens* |  | 55 | 0.04 | MS | 4.82 ± 0.12 |  |
| Greater pipefish | *Syngnathus acus* |  | 41 | 0.03 | ES | 3.60 ± 0.15 |  |
| Reticulated dragonet | *Callionymus reticulatus* |  | 36 | 0.03 | MS | 4.74 ± 0.13 |  |
| Painted goby | *Pomatoschistus pictus* |  | 23 | 0.02 | MS | 5.00 ± 0.00 |  |
| Dover Sole | *Solea solea* |  | 21 | 0.02 | MM | 4.13 ± 0.06 |  |
| Whiting | *Merlangius merlangus* |  | 18 | 0.01 | MM | 4.26 ± 0.11 |  |
| Deep Snouted Pipefish | *Syngnathus typhle* |  | 12 | 0.01 | ES | 3.29 ± 0.14 |  |
| Goby sp. |  |  | 11 | 0.01 | n/a |  |  |
| Mackerel | *Scomber scombrus* |  | 10 | 0.01 | ES | 4.96 ± 0.04 |  |
| Short-spined Sea Scorpion | *Myoxocephalus scorpius* |  | 9 | 0.01 | MM | 4.10 ± 0.17 |  |
| Solenette | *Buglossidium luteum* |  | 9 | 0.01 | MS | 5.00 ± 0.00 |  |
| Lesser weaver | *Echiichthys vipera* |  | 8 | 0.01 | MS | 5.00 ± 0.00 |  |
| Common dragonet | *Callionymus lyra* |  | 7 | 0.01 | MS | 5.00 ± 0.00 |  |
| Transparent goby | *Aphia minuta* |  | 7 | 0.01 | ES | 3.70 ± 0.19 |  |
| Worm Pipefish | *Nerophis lumbriciformis* |  | 7 | 0.01 | ES | 3.90 ± 0.31 |  |
| Other taxa^1^ |  |  | 25 |  |  |  |  |

^1^Species that accounted for less than 5 individuals overall have been combined into “other taxa”, comprising of *Anguilla anguilla, Sparus aurata, Ciliata mustela, Scophthalmus rhombus, Mullus surmuletus, Salmo trutta, Gasterosteus aculeatus, Trisopterus luscus, Abramis brama, Syngnathus rostellatus, Liparis montagui, Parablennius gattorugine, Chelidonichthys lucerna, Alosa fallax.*
